# Isolation and Genomic Characterization of a Duck-Origin GPV-Related Parvovirus from Cherry Valley Ducklings in China

**DOI:** 10.1371/journal.pone.0140284

**Published:** 2015-10-14

**Authors:** Hao Chen, Yanguo Dou, Yi Tang, Zhenjie Zhang, Xiaoqiang Zheng, Xiaoyu Niu, Jing Yang, Xianglong Yu, Youxiang Diao

**Affiliations:** 1 College of Animal Science and Technology, Shandong Agricultural University, Tai’an, Shandong, 271018, China; 2 College of Basic Medicine, Taishan Medical University, Tai’an, Shandong, 271000, China; Oklahoma State University, UNITED STATES

## Abstract

A newly emerged duck parvovirus, which causes beak atrophy and dwarfism syndrome (BADS) in Cherry Valley ducks, has appeared in Northern China since March 2015. To explore the genetic diversity among waterfowl parvovirus isolates, the complete genome of an identified isolate designated SDLC01 was sequenced and analyzed in the present study. Genomic sequence analysis showed that SDLC01 shared 90.8%–94.6% of nucleotide identity with goose parvovirus (GPV) isolates and 78.6%–81.6% of nucleotide identity with classical Muscovy duck parvovirus (MDPV) isolates. Phylogenetic analysis of 443 nucleotides (nt) of the fragment A showed that SDLC01 was highly similar to a mule duck isolate (strain D146/02) and close to European GPV isolates but separate from Asian GPV isolates. Analysis of the left inverted terminal repeat regions revealed that SDLC01 had two major segments deleted between positions 160–176 and 306–322 nt compared with field GPV and MDPV isolates. Phylogenetic analysis of Rep and *VP1* encoded by two major open reading frames of parvoviruses revealed that SDLC01 was distinct from all GPV and MDPV isolates. The viral pathogenicity and genome characterization of SDLC01 suggest that the novel GPV (N-GPV) is the causative agent of BADS and belongs to a distinct GPV-related subgroup. Furthermore, N-GPV sequences were detected in diseased ducks by polymerase chain reaction and viral proliferation was demonstrated in duck embryos and duck embryo fibroblast cells.

## Introduction

Waterfowl parvoviruses cause high morbidity and mortality in goslings and Muscovy ducklings, with mortality rates between 10% and 80% and even up to 95%[1]. Most goose parvovirus (GPV) and Muscovy duck parvovirus (MDPV) isolates are virulent and pathogenic to young animals[2]. This disease is characterized by anorexia, prostration, watery diarrhea, enteric symptoms, and death. Survived young birds and infected older birds show degenerative skeletal muscle myopathy and growth retardation[3–5]. The disease, known as Derzsy’s disease, causes great economic losses in waterfowl husbandry. In addition, some distinct GPV strains cause symptoms such as short bills with protruding tongues and growth retardation in mule and Tsaiya ducks in France, Hungary, Poland, and Taiwan[6–9]. The latter disease has been named beak atrophy and dwarfism syndrome (BADS) based on the typical symptoms.

Waterfowl parvoviruses are members of the genus *Anseriform dependoparvovirus 1* in the family *Parvoviridae* and contain a linear, single-stranded DNA genome of about 5 kb in length[10]. The genome contains two major open reading frames (ORFs): the left ORF that encodes for the regulatory (*Rep*) protein, and the right ORF that encodes for three capsid proteins, VP1, VP2, and VP3[11, 12]. The latter two proteins are generated by differential splicing and share the same carboxy-terminal portion with VP1[13, 14]. The capsid proteins play important roles in virus tropism, host range, and pathogenicity[15, 16]. To date, however, there has been no report on the genomic sequence and analysis of the duck parvovirus that is the causative agent of BADS.

Since March 2015, a moderately pathogenic novel GPV-related parvovirus (N-GPV) has been reported in commercial Cherry Valley duck flocks in Northern China. The morbidity among ducks with typical symptoms of BADS has been 10% to 30% and even up to 50%. The diseased ducks also displayed other clinical symptoms including a swollen tongue, watery diarrhea, shorter tibia, slightly depauperate liver, and swelling and hemorrhage in the thymus. A total of 37 visceral liver and 37 cloacal swab samples were collected from four diseased commercial Cherry Valley duck flocks in four herds (Gaotang, Dangshan, Peixian and Zoucheng counties) in Northern China. Forty cloacal swab samples were collected from healthy ducks in the diseased flocks. The diseased ducks observed showed beak atrophy, tongue protrusion, and growth retardation. Polymerase chain reaction (PCR) analysis confirmed that a novel parvovirus was the causative agent of the disease. Livers of diseased ducks from four different herds were used for virus isolation, and the full-length genome of SDLC01 was sequenced. Based on a 661-base segment of the VP3 genes of GPV, the GPV-related virus was confirmed to be the causative agent of the disease that quickly spread to other herds in Northern China.

In this study, a distinct GPV-related duck parvovirus strain was isolated from a diseased Cherry Valley duckling in Shandong province of China in 2015 and designated SDLC01. To further explore the genetic diversity of the waterfowl parvovirus, complete genomic sequencing of the SDLC01 strain and phylogenetic comparison with classical waterfowl parvovirus isolates and vaccine strains were performed. Furthermore, we examined the ability of the virus to replicate in duck and goose embryos and in duck embryo fibroblast (DEF) cells. The nucleotide sequences of the genome and of the inverted terminal repeat (ITR) were aligned with the sequences of other GPV/MDPV strains. The amino acid sequences of Rep and VP1 of the isolate were fully analyzed.

## Materials and Methods

### Ethics Statement

The sample collection procedures were approved by the Animal Care and Use Committee of Shandong Agricultural University and performed in accordance with the “Guidelines for Experimental Animals” of the Ministry of Science and Technology (Beijing, China). All efforts were made to minimize suffering. The diseased ducks were euthanized in CO_2_ before sample collection. No specific permissions were required for these locations/activities. There are not endangered or protected species involved in these locations.

### Virus isolation

The liver samples were homogenized in phosphate buffered saline (PBS, pH 7.2), freeze-thawed three times, and centrifuged at 8000 × *g* for 15 min. The supernatant was filtered through a 0.22 μm filter and the filtrate was inoculated into the chorioallantoic cavity of 9-day-old duck embryos and 11-day-old goose embryos (0.2 mL/embryo), respectively. The embryos were monitored daily for 7 days, and no deaths were observed among the embryos after three passages. The embryos showed growth retardation and slight hemorrhage. DEF cells were inoculated with pooled allantoic fluids (1:10 dilution), grown for eight passages and monitored daily for cytopathic effects. The culture supernatants were harvested at 5–7 days post-inoculation and stored at −70°C as a viral stock.

### Indirect fluorescent antibody assay (IFA)

The infected DEF cells were incubated for an additional 72 h, washed with PBS three times, and loaded onto microscope slides, which were fixed in a chilled acetone and methanol mixture (1:1) at −20°C for 10 min and washed three times prior usage. The cells on the slides were covered with N-GPV-specific polyclonal antibodies (mouse antiserum against the duck parvovirus), which was diluted in PBS (1:100), and incubated at 37°C for 1 h. The slides were gently washed and then treated with sufficient fluorescein isothiocyanate-labeled goat anti-mouse IgG (BoAoSeng Company, Beijing, China). The slides were incubated at 37°C for 1 h. Finally, the slides were mounted with glycerol buffer and observed using a fluorescence microscope (Olympus, Tokyo, Japan).

### Amplification of the duck parvovirus genome

The liver homogenates and cloacal swabs were prepared for PCR using a QIAamp DNA mini kit (Qiagen, Hilden, Germany). All samples were subjected to polymerase chain reaction (PCR) using specific primers (VP3-F: 5′-GAGCATCA -ACTCCCGTATGTCC-3′ and VP3-R: 5′-CTACTTCCTGCTCGTCCGTGA-3′) (433~1093bp) to amplify a partial sequence (661 bp) of the N-GPV VP3, based on the VP3 gene sequence of a classical GPV (KC996729). All the samples were N-GPV-positive, and the PCR products from different herds were purified and sequenced. On the basis of significant scores obtained using the basic local alignment search tool (BLAST), all these amplified PCR fragments showed 99% similarity with the sequence of a goose parvovirus, 82-0321v(GenBank Accession no. EU583389), isolated in Taiwan[17]. The complete genome of SDLC01 was sequenced after PCR using primers designed based on the conserved regions in the genome of GPV isolate 82-0321v (Table 1). PCR conditions were as follows: 95°C for 5 min, 30 cycles of denaturation (95°C for 30 s), annealing (55°C for 1 min), and extension (72°C for 2 min), followed by a final extension at 72°C for 10 min. The primer (P1-R, P2-F and P5-R) annealing sites were located in the middle loop (bubble) regions of the inverted terminal repeats in the 5′ and 3′ non-coding regions, respectively. The full-length genomic sequence was assembled using five overlapping DNA fragments.

**Table 1:** Primers used to amplify the complete nucleotide sequence of N-GPV isolate SDLC01.

Primers	Nucleotide sequence (5’-3’)	Position	Length of products (bp)
**P1-F**	CTTATTGGAGGGTTCGTT	1–18	176
**P1-R**	GCATGCGCGTGGTCAACCTAACA	154–176	
**P2-F**	GCATGCCGCGCGGTCAGCCCAAT	171–193	1389
**P2-R**	AGGGTACAGCATGGACAATAG	1539–1559	
**P3-F**	CACCACCGGGAAGACCAACAT	1510–1530	962
**P3-R**	CAGCTTTCAGATTCCGCCACG	2451–2471	
**P4-F**	TCGTGGCGGAATCTGAAAGC	2450–2469	1752
**P4-R**	CATTCCTGGTAAAGCTCCAAGAAC	4178–4201	
**P5-F**	CATACAGATCTGGCAGCACTACAGCA	4020–4045	835
**P5-R**	CTGTTAGGTTGACCACGCGCATGC	4831–4854	

The underlined sequence “GCATGC” means *Sph* I restriction site, in the “bubble” region of ITR.

### Cloning and sequencing

The amplified PCR products were purified using a gel extraction kit (Takara, Dalian, China), cloned into the pMD18-T vector (Takara, Dalian, China) and transformed into *E*.*coli* DH5α competent cells. Clones were sequenced by BGI Tech (BGI Tech, Shenzhen, China). To ensure the accuracy of the genomic sequence, duplicate clones of the five fragments were sequenced. The five fragments were assembled to build a full-length complete genomic sequence of SDLC01 using the DNASTAR program (Madison, WI, USA). Partial VP3 gene sequences (661 bp) of other isolates from the four herds were also obtained. To analyze the genetic diversity of waterfowl parvoviruses, 443 bp of partial VP1 genes (nucleotides 163–657) of GPV were amplified and sequenced using the method previously described[18].

### Sequence analysis

The obtained sequences were compared with those in the GenBank database using BLASTP searches. Nucleotide and amino acid sequences were aligned using the MegAlign software included in the DNASTAR package 5.0, using the ClustalW matrix as the comparison scoring table. Phylogenetic trees of the aligned nucleotide and amino acid sequences were constructed using the neighbor-joining method included in the MEGA 5.0 version, based on 1000 bootstrap replications[19]. The reference waterfowl parvovirus isolates are listed in Table 2. The GenBank accession number for the sequence determined in this study is SDLC01 (KT343253).

**Table 2:** Description of waterfowl parvovirus isolates involved in this study.

GPV isolates	Accession no.	Pathogenicity	Host	Separatum
**SHM319**	U34761	Pathogenic	Goose	German
**VG32/1**	EU583392	Vaccine	Goose	German
**D146/02**	AY496906	Moderately pathogenic	Mule duck	France
**Hoekstra**	AY496907	Vaccine	Goose	France
**FM**	NC_006147	Pathogenic	Muscovy duck	France
**Pusztafoldvar/79**	AY496903	Low pathogenic	Goose	Hungary
**Csongrad/80**	AY496902	Low pathogenic	Goose	Hungary
**B**	U25749	Pathogenic	Goose	Hungary
**82–0308**	AY382883	Pathogenic	Goose	Taiwan
**82-0321v**	EU583389	Vaccine	Goose	Taiwan
**SHFX1201**	KC478066	Pathogenic	Swan	China
**Y**	KC178571	Pathogenic	Muscovy duck	China
**YZ99-6**	KC996730	Pathogenic	Goose	China
**GD**	AY512830	Pathogenic	Goose	China
**SYG61v**	KC996729	Vaccine	Goose	China
**SH**	JF333590	Pathogenic	Goose	China
**SAAS-SHNH**	KC171936	Pathogenic	Muscovy duck	China
**D**	JF926696	Pathogenic	Muscovy duck	China
**MDPV-GX5**	KM093740	Pathogenic	Muscovy duck	China

## Results

### Identification of a duck parvovirus

**Fig 1:**
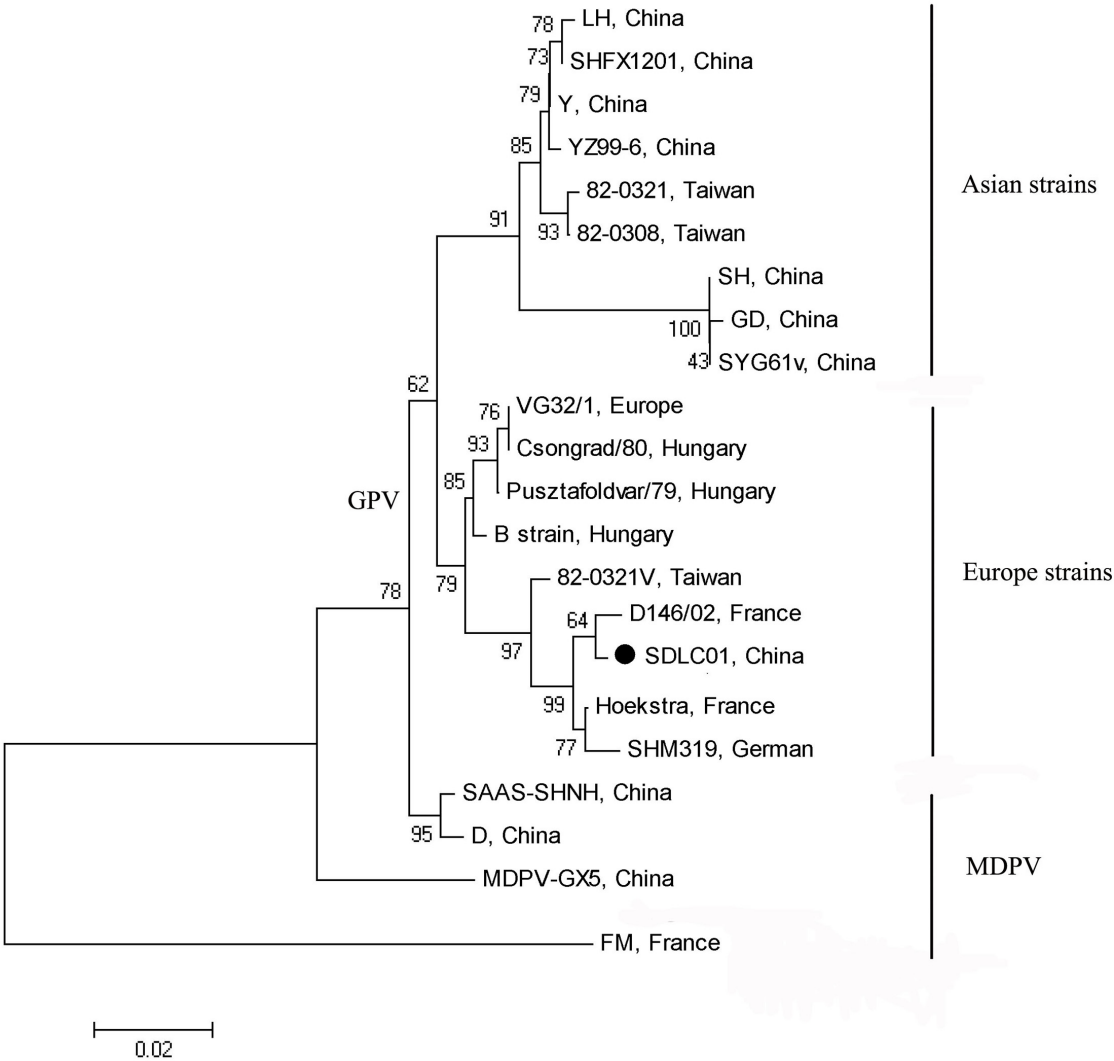
Phylogenetic tree constructed using the neighbor-joining method, based on the sequence of the fragment A of SDLC01 (●) and other waterfowl parvovirus isolates available in GenBank database. The consensus tree from 1000 bootstrap replicates is shown. The percentage of trees that contained the consensus branch is also shown for each branch. The scale at the bottom shows the number of substitutions inferred per site.

Using the GPV VP3-based PCR method, DNA fragments of approximately 661 bp were produced from the 114 samples collected. BLASTP searches revealed that the amplicons were most closely related to the partial VP3 gene of GPV attenuated isolate 82-0321v. Four N-GPV isolates (designated SDLC01, SDLY01, JSXU01, and AHSZ01) from this study shared completely identical sequences of 661 bp with each other. To further investigate the relationship of the four isolates to other waterfowl parvoviruses, a phylogenetic tree was constructed based on alignment of 443-nucleotide (nt) VP1 gene sequences (Fig 1). The tree demonstrated that the SDLC01 strain from this study was clustered into a subgroup of European strains within the GPV clade. SDLC01 was closest to the D146/02 strain, which was isolated from a diseased mule duck with BADS in France[7]. The partial VP3 gene sequences of the four N-GPV isolates shared 91.4%–99.5% identity at DNA level and 93.9%–98.6% identity at the amino acid level with goose parvovirus isolates, and 78.1%–79.0% identity at the DNA level and 79.6%–82.3% identity at the amino acid level with Muscovy duck parvovirus isolates. These results suggested that the four N-GPV isolates from this study likely represented a GPV-related virus.

### Virus isolation and propagation

**Fig 2:**
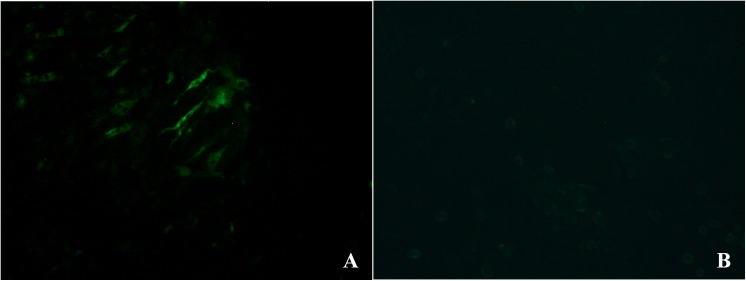
Detection of viral protein expression in DEF cells infected with N-GPV. (A) DEF cells infected with SDLC01 (B) negative control was fixed at 24 h post-infection and examined with mouse antiserum against the duck parvovirus.

After chorioallantoic membrane inoculation of the liver homogenate and propagation for three passages, no embryo deaths occurred on the third passage until 7 dpi, and the allantoic fluids were harvested for PCR assay. The chorioallantoic membrane from the infected duck embryos looked thicker and grayer, while no obvious changes were observed in infected goose embryos. PCR results showed that viral DNA was present both in duck embryos and allantoic fluids, but not in goose embryos and allantoic fluids, after three passages. DEF cultures were inoculated with preparations obtained from the embryos to propagate virus isolates for three passages. Cytopathic effects occurred after 7 passages. Green fluorescence signals were detected in DEFs at 72 h post-infection with SDLC01, but not in goose embryo fibroblasts (GEFs) (Fig 2). As the isolate could not be well propagated during the initial three passages, a viral stock was stably maintained in duck embryos and DEF cells for additional passages.

### Analysis of full-length genomic sequences

**Fig 3:**
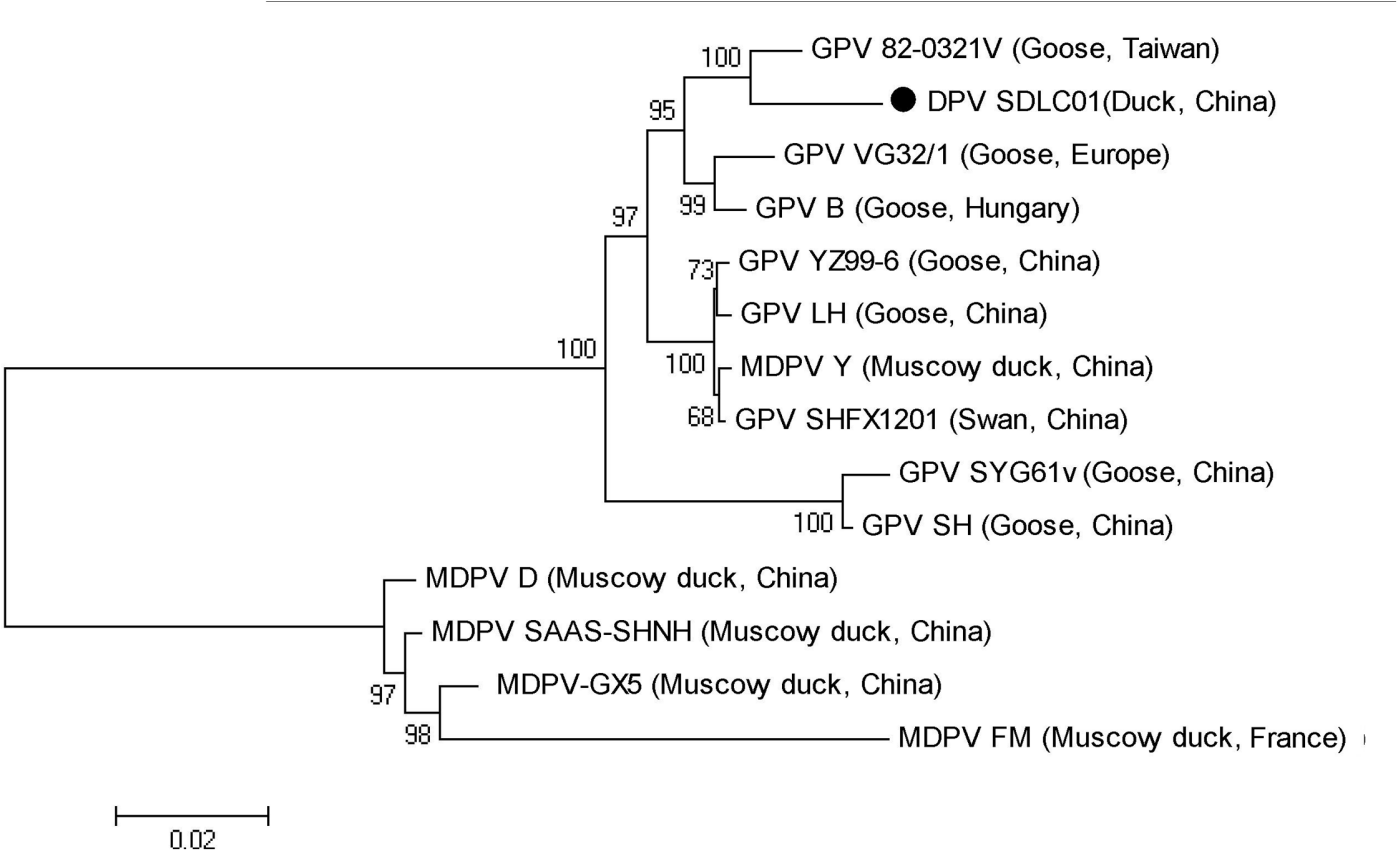
Phylogenetic relationship between the SDLC01 (●) and other waterfowl parvovirus isolates available in GenBank database based on the complete genomic sequences in the phylogenetic tree, built using the neighbor-joining method. Numbers at nodes indicate bootstrap percentages obtained using 1000 replicates.

The sequencing results showed that the full-length genome of SDLC01 was 5,006 nucleotides in length. The left ORF (nt 497–2,380) encoded the nonstructural protein (*Rep*) of 627 amino acids, and the right ORF (nt 2,399–4,597) encoded the VP1 protein of 732 amino acids. The start codons of VP2 and VP3 were located at 2,834 and 2,993 nt, respectively. The ITRs consisted of 366 nt in both 3′ and 5′ non-coding regions of the SDLC01 genome. Alignment of the ITR sequences of two MDPV isolates, two GPV isolates and SDLC01, and their comparison with the consensus sequence showed that the SDLC01 strain had three short fragments deleted, compared with GPV attenuated isolate 82-0321v, and two short fragments deleted, compared with the MDPV isolates. There were two inserted fragments in SDLC01 and attenuated vaccine strain VG32/1, which were absent in the other three parvovirus isolates (Fig 3). The phylogenetic tree displayed that SDLC01 was clustered into a branch with two isolates (VG32/1 and B) from Europe and an isolate (82-0321v) from Taiwan. However, the SDLC01 genome shared the highest identity (96.8%) with the 82-0321v strain, which is an attenuated GPV isolated in Taiwan in 1982. The SDLC01 strain shared 90.8%–94.6% homology with other reference GPVs and 78.6%–81.6% homology with classical MDPV isolates.

### Phylogenetic analysis of NS and VP1 genes

**Fig 4:**
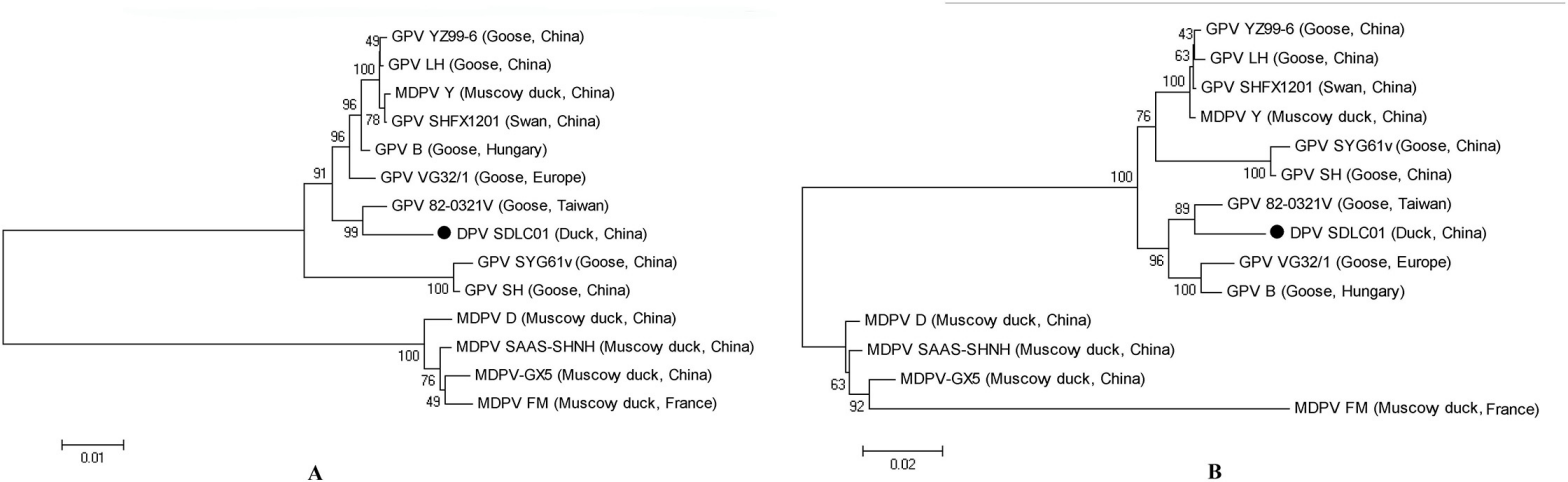
Phylogenetic relationships between the SDLC01 isolate (●) in this study and other waterfowl parvovirus isolates in the phylogenetic tree, built using the neighbor-joining method. The analyses were based on nucleotide sequences of NS (A) and VP1genes (B).

To determine the genetic diversity between SDLC01 and other GPV and MDPV isolates, phylogenetic trees of NS and VP1 genes of waterfowl parvoviruses were constructed (Fig 4). Phylogenetic analysis of NS genes showed that the SDLC01 strain had the highest homology to GPV vaccine strain 82-0321v. NS gene of SDLC01 shared 93.6%–97.8% identity with the genes of other GPV strains and 82.1%–83.5% identity with the genes of MDPV strains. The phylogenetic tree based on VP1 gene sequences showed that the SDLC01 strain was clustered, together with vaccine strains 82-0321v and VG32/1 and the standard B strain, into a distinct clade included in the GPV-related lineage, compared with other GPVs isolated in China. The VP1 genes of SDLC01 and other strains were aligned using BLASTN. The results showed that SDLC01 was highly similar (≥98% similarity) to GPV isolates: SHM319 (99%), 82-0321v (98%) and 82–0408 (98%) (data not shown). The VP1 gene of SDLC01 showed 93.2%–97.6% nucleotide sequence identity with the genes of other GPV isolates and 81.3%–88.7% identity with the genes of MDPV isolates.

### Amino acid sequence analysis of Rep

**Fig 5:**
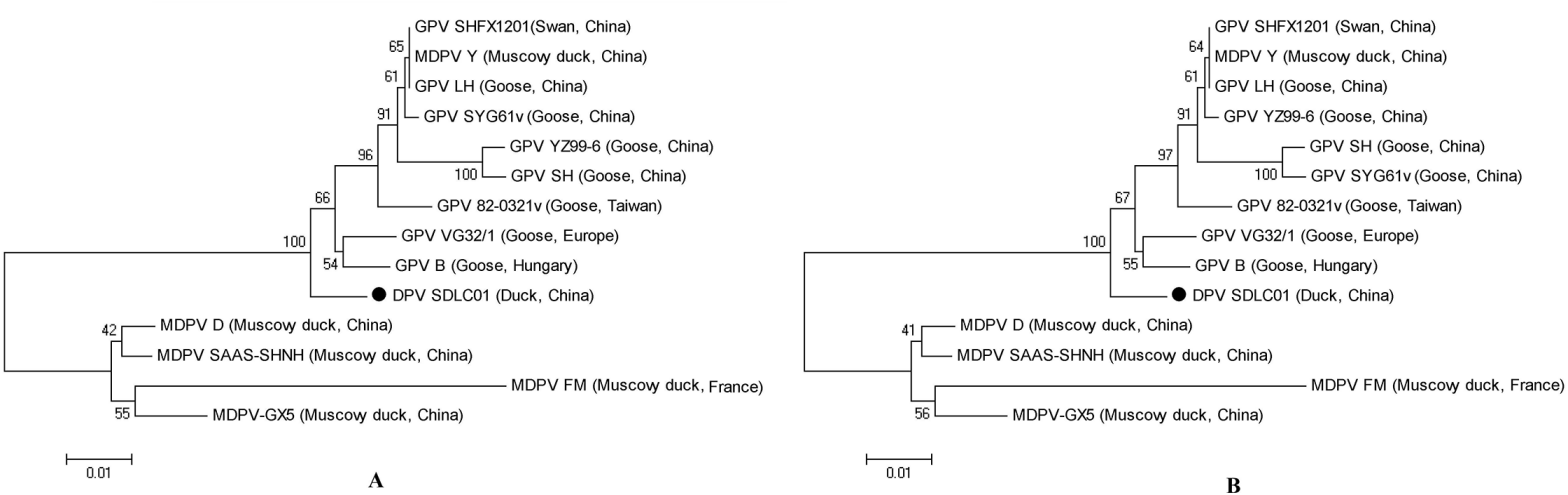
Phylogenetic relationships between the SDLC01 isolate (●) in this study and other waterfowl parvovirus isolates in the phylogenetic tree, built using the neighbor-joining method. The analyses were based on amino acid sequences of Rep (A) and VP1proteins (B).

The amino acid sequences of the Rep proteins were predicted from the nucleotide sequences of NS genes. The Rep sequences of GPV isolates 82-0321v, VG32/1, SYG61v, and the virulent B strain were aligned with that of SDLC01. Compared with the four GPV isolates, the Rep protein of SDLC01 had 14 amino acid substitutions (I50T, L89M, K131R, A140S, A350V, V468I, E498R, R553K, N555T, R556C, E573Q, D594Y, K605T, and V617A). However, no changes were detected in the motifs involved in NTP binding (336–343 amino acids) and DNA binding (142–168 amino acids) of the Rep protein[20, 21]. Phylogenetic analysis of the deduced amino acid sequences of NS genes showed that SDLC01 was 96.2%–97.6% and 89.3%–89.8% identity to GPV and MDPV isolates, respectively. The phylogenetic tree demonstrated that waterfowl parvoviruses were divided into two groups (GPV and MDPV) (Fig 5A). SDLC01 was clustered into a single-member subgroup in the GPV group, whereas the GPV isolates were clustered together into another subgroup. These results indicated that the SDLC01 strain showed a close genetic relationship with the GPV isolates, but was clustered in a distinct subgroup.

### Amino acid sequence analysis of VP1

The amino acid sequences of the VP1 proteins were predicted from the nucleotide sequences. Compared with GPV isolates 82-0321v, VG32/1, SYG61v, and the virulent B strain, the VP1 protein of SDLC01 had eight amino acid substitutions (Q89L, D114H, Q116H, D142E, A180V, S450N, S498N, and H660N). The same three amino acids (142E, 498N, and 660N) were also present in four MDPV isolates, D, FM, GX-5, and SAAS-SHNH. Only the residue 498N found in VP1 protein of SDLC01 was located in the putative receptor binding site of the VP1 protein of GPV (471–620 amino acids) [16, 22]. Phylogenetic analysis of the deduced amino acid sequences of the VP genes showed that SDLC01 was 96.3–98.2% and 87.7–92.6% homologous to GPV and MDPV isolates, respectively. The phylogenetic tree of VP1 proteins was similar to that of the *Rep* proteins (Fig 5B). SDLC01 was clustered into a distinct subgroup included in the GPV-related virus lineage. These results showed that SDLC01 might be a unique GPV-related parvovirus isolated from a Cherry Valley duck.

## Discussion

In the present study, we detected parvovirus sequences in diseased ducks with BADS from different herds in Northern China. The disease has previously been reported in France, Poland, and Taiwan[9, 23, 24]. The genome sequences of GPV and MDPV isolates have been reported, but only partial sequences of N-GPV isolates D146/02, D176/02 (France) and 90219 (Taiwan) were available in the NCBI database. However, the full-length genome of the BADS causative agent was sequenced and analyzed for the first time in this study. Our sequence analysis indicated that the four N-GPV isolates were closely related to each other and confirmed them to be novel members of the family *Parvoviridae*. All samples from healthy ducks in the infected flocks were found to be GPV-positive in phylogenetic analysis using PCR, indicating that N-GPV had a powerful capacity for horizontal transmission. Field cases occurred early in 13-day-old ducks, which corresponded to the number of days after an accident in 1-day-old ducklings challenged with SDLC01 via intramuscular injection (unpublished data). The isolates were determined to be moderately pathogenic based on the clinical pathological findings in the field. A further study is necessary to confirm the ability of vertical transmission of N-GPV. The isolates could be propagated in duck embryos and DEF cells, but not in goose embryos or GEF cells. It is not clear whether Muscovy duck embryos or embryo fibroblast cells could be used for viral proliferation.

The complete genome sequence of isolate SDLC01 consisted of 5,006 nt and showed the highest nucleotide sequence similarity (96.8%) with GPV attenuated vaccine strain 82-0321v derived from virulent 82–0321 isolated in Taiwan[22]. SDLC01 had a shorter ITR of 366 nt (78 nt shorter compared with classical GPV isolate B and 15 nt shorter compared with 82-0321v). The SDLC01 isolate examined in this study is genetically closely related to GPV isolates, with 90.8%–94.6% of nucleotide similarity at the whole-genome level. Compared with pathogenic GPV and MDPV isolates, 82-0321v and SDLC01 had two major deletion regions (160–176 nt and 306–322 nt). The deletion phenomenon therefore seems to be correlated with virulence, even though the three transcription factor binding sites (E-box) and a putative Rep recognition sequence (GTTC) were located in the deleted region[20, 21, 25]. Specific changes (host range, titers, and growth characteristics) of N-GPV, induced by other deletions and nucleotide mutations, are yet to be elucidated.

Genetic analysis and a phylogenetic study of the 443-nt fragment B were more useful for determining the divergence of the strains, compared with the fragment A[18]. In this study, the SDLC01 strain showed a very high similarity (99.5%) to D146/02 isolated from a mule duck in France. Phylogenetic analysis of SAAS-SHNH strain provided evidence of genetic recombination events between MDPVs and GPVs[26]. Interestingly, SDLC01 was clustered within the European strain subgroup, which included pathogenic isolates (B strain and SHM319) [3, 27], a moderately pathogenic isolate (D146/02), low pathogenic isolates (Pusztafoldvar/79 and Csongrad/80) and vaccine isolates (VG32/1 and Hoekstra) [18]. Other GPV strains isolated in China mainland and Taiwan were clustered in the Asian strain subgroup[13]. This suggests that SDLC01 may have had a common ancestor with European GPV isolates.

The phylogenetic tree created from the Rep protein amino acid sequences was in agreement, except for a slight difference, with that created from the VP1 protein amino acid sequences. Both demonstrated that the SDLC01 isolate was closely related with GPV vaccine strain VG32/1 and the virulent B strain, and formed a distinct lineage from the previously identified GPV isolates. These results indicate that the SDLC01 strain may be a unique waterfowl parvovirus that shows a closer genetic relationship with European GPV strains than with Asian strains.

**Fig 6:**
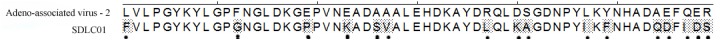
Sequence alignments of Parvovirus PLA_2_ Motifs and sPLA_2_ representatives between SDLC01 strain and Adeno-associated virus -2.

The Rep proteins are functional in the control of gene expression, DNA replication, and genome encapsidation[28]. VP1 and VP2 differ from VP3 only by N-terminal extensions of 65 amino acids, common to VP1 and VP2, and by further 137 amino acids which are unique for VP1[29]. Adeno-associated virus type 2 (AAV2) has a close genetic relationship with waterfowl parvoviruses (MDPV and GPV)[30]. VP1 analysis in AAV2 capsid mutants confirmed the presence of viral phospholipase A_2_ (PLA_2_) activity and highlighted the importance of this enzymatic activity in the virus life cycle[31]. Deletion or mutation of critical amino acid residues in the putative parvoviral PLA_2_ in VP1 leads to strongly reduced infectivity in *Porcine parvovirus*, even though capsid assembly and packaging of DNA are not impaired[32, 33]. This decreased infectivity can be partially attributed to the loss of the PLA_2_ catalytic domain and its activity. Suppression of PLA_2_ induces the disturbance of calcium and phosphorus metabolism in ducks, causing osteodysplasty, particularly in beak, metatarsus, and wing bones. Alignment of the amino acid sequences of the PLA_2_ domain demonstrated that the SDLC01 strain had a mutation in residue 32, with Asn substituting for the Ser residue present in other GPV isolates (Fig 6). The mutation in PLA_2_ possibly plays an important role in viral infectivity.

In the present study, a DPV isolate associated with a BADS outbreak in ducks in Northern China was obtained, sequenced, and characterized. The SDLC01 strain is close to European GPV isolates, but separated from Asian GPV isolates. It is clear from the multiple sequence alignments and phylogenetic analysis that SDLC01 represents a distinct member of the GPV-related parvoviruses. These results suggest that deletions in ITR and mutations in Rep and VP1 may play significant roles in viral infectivity, host range, and pathogenicity. Epidemiological characterization of N-GPV is necessary to prevent and control BADS.
